# Freshwater phytoplankton diversity: models, drivers and implications for ecosystem properties

**DOI:** 10.1007/s10750-020-04332-9

**Published:** 2020-07-04

**Authors:** Gábor Borics, András Abonyi, Nico Salmaso, Robert Ptacnik

**Affiliations:** 1 Department of Tisza Research, Centre for Ecological Research, Danube Research Institute, Bem tér 18/c, 4026 Debrecen, Hungary; 2 GINOP Sustainable Ecosystems Group, Centre for Ecological Research, Klebelsberg Kuno u. 3, 8237 Tihany, Hungary; 3 Centre for Ecological Research, Institute of Ecology and Botany, Alkotmány u. 2-4, 2163 Vácrátót, Hungary; 4 WasserCluster Lunz – Biologische Station GmbH, Dr. Carl Kupelwieser-Promenade 5, 3293 Lunz am See, Austria; 5 Research and Innovation Centre, Fondazione Edmund Mach, Via E. Mach 1, 38010 San Michele all’Adige, Italy

**Keywords:** Community assembly, Diversity maintenance, Ecosystem functioning, Functional diversity, Molecular approaches, Taxonomic diversity

## Abstract

Our understanding on phytoplankton diversity has largely been progressing since the publication of Hutchinson on the paradox of the plankton. In this paper, we summarise some major steps in phytoplankton ecology in the context of mechanisms underlying phytoplankton diversity. Here, we provide a framework for phytoplankton community assembly and an overview of measures on taxonomic and functional diversity. We show how ecological theories on species competition together with modelling approaches and laboratory experiments helped understand species coexistence and maintenance of diversity in phytoplankton. The non-equilibrium nature of phytoplankton and the role of disturbances in shaping diversity are also discussed. Furthermore, we discuss the role of water body size, productivity of habitats and temperature on phytoplankton species richness, and how diversity may affect the functioning of lake ecosystems. At last, we give an insight into molecular tools that have emerged in the last decades and argue how it has broadened our perspective on microbial diversity. Besides historical backgrounds, some critical comments have also been made.

**Electronic supplementary material:**

The online version of this article (10.1007/s10750-020-04332-9) contains supplementary material, which is available to authorized users.

## Introduction

Phytoplankton is a polyphyletic group with utmost variation in size, shape, colour, type of metabolism, and life history traits. Due to the emerging knowledge in nutritional capabilities of microorganisms, our view of phytoplankton has drastically changed (Flynn et al., 2013). Phagotrophy is now known from all clades except diatoms and cyanobacteria. At the same time, ciliates, which have not been considered as part of ‘phytoplankton’, span a gradient in trophic modes that render the distinction between phototrophic phytoplankton and heterotrophic protozoa meaningless. This complexity has been expressed in the high diversity of natural phytoplankton assemblages. Diversity can be defined in many different ways and levels. Although the first diversity measure that encompassed the two basic components of diversity (i.e., the number of items and their relative frequencies) appeared in the early forties of the last century (Fisher et al., 1943), in phytoplankton ecology, taxonomic richness has been used the most often as diversity estimates. Until the widespread use of the inverted microscopes, phytoplankton ecologists did not have accurate abundance estimation methods and the net plankton served as a basis for the analyses. Richness of taxonomic groups of net samples, and their ratios were used for quality assessment (Thunmark, 1945, Nygaard, 1949).

The study of phytoplankton diversity received a great impetus after Hutchinson’s (1961) seminal paper on the paradox of the plankton. The author not only contrasted Hardin’s competitive exclusion theory (Hardin, 1960) with the high number of co-occurring species in a seemingly homogeneous environment, but outlined possible explanations. He argued for the non-equilibrium nature of the plankton, the roles of disturbances and biotic interactions, moreover the importance of benthic habitats in the recruitment of phytoplankton. The ‘paradox of the plankton’ largely influenced the study of diversity in particular and the development of community ecology in general (Naselli-Flores & Rossetti, 2010). Several equilibrium and non-equilibrium mechanisms have been developed to address the question of species coexistence in pelagic waters (reviewed by Roy & Chattopadhyay, 2007). The paradox and the models that aimed to explain the species coexistence in the aquatic environment have been extended to terrestrial ecosystems (Wilson, 1990). Wilson reviewed evidences for twelve possible mechanisms that potentially could explain the paradox for indigenous New Zealand vegetation, and found that four of them, such as gradual climate change, cyclic successional processes, spatial mass effect and niche diversification, were the most important explanations. By now, the paradox has been considered as an apparent violation of the competitive exclusion principle in the entire field of ecology (Hening & Nguyen, 2020).

**Fig. 1:**
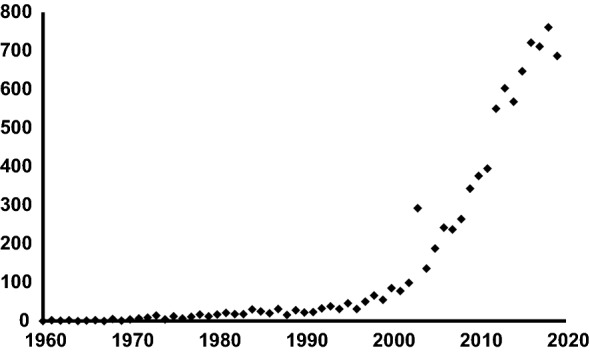
Annual number of hits on Google Scholar for the keywords “phytoplankton diversity”

Although Hutchinson’s contribution (Hutchinson, 1961) has given a great impetus to research on species coexistence, the number of studies on phytoplankton diversity that time did not increase considerably (Fig. 1), partly because in this period, eutrophication studies dominated the hydrobiological literature.

Understanding the drivers of diversity has been substantially improved from the 70 s when laboratory experiments and mathematical modelling proved that competition theory or intermediate disturbance hypothesis (IDH) provided explanations for species coexistence. Many field studies also demonstrated the role of disturbances in maintaining phytoplankton diversity, and these results were concluded by Reynolds and his co-workers (Reynolds et al., 1993).

From the 2000 s a rapid increase in phytoplankton research appeared (Fig. 1), which might be explained by theoretical and methodological improvements in ecology. The functional approaches—partly due to Colin Reynolds’s prominent contribution to this field (Reynolds et al., 2002)—opened new perspectives in phytoplankton diversity research. Functional trait and functional ‘group’-based approaches have gained considerable popularity in recent years (Weithoff, 2003; Litchman & Klausmeier, 2008; Borics et al., 2012; Vallina, et al., 2017; Ye et al., 2019).

Analysis of large databases enabled to study diversity changes on larger scales in lake area, productivity or temperature (Stomp et al., 2011). Recent studies on phytoplankton also revealed that phytoplankton diversity was more than a single metric by which species or functional richness could be described, instead, it was an essential characteristic, which affects functioning of the ecosystems, such as resilience (Gunderson 2000) or resource use efficiency (Ptacnik et al., 2008; Abonyi et al., 2018a, b).

The widespread use of molecular tools that reorganise phytoplankton taxonomy and reveal the presence of cryptic diversity, has changed our view of phytoplankton diversity. In this study, we aim to give an overview of the above-mentioned advancements in phytoplankton diversity. Here we focus on the following issues:

- measures of diversity,
- mechanisms affecting diversity,
- changes of diversity along environmental gradients (area, productivity, temperature),
- the functional diversity–ecosystem functioning relationship, and
- phytoplankton diversity using molecular tools.

More than eight thousand studies have been published on “phytoplankton diversity” since the term first appeared in the literature in the middle of the last century (Fig. 1), therefore, in this review we cannot completely cover all the important developments made in recent years. Instead, we focus on the most relevant studies considered as milestones in the field, and on the latest relevant contributions. This study is a part of a Hydrobiologia special issue dedicated to the memory of Colin S. Reynolds, who was one of the most prominent and influential figures of phytoplankton ecology in the last four decades, therefore, we have placed larger emphasis on his concepts that helped our understanding of assembly and diversity of phytoplankton.

### Measures of diversity

In biology, the term “diversity” encompasses two basic compositional properties of assemblages: species richness and inequalities in species abundances. Verbal definitions of diversity cannot be specific enough to describe both aspects, but these can be clearly defined by the mathematical formulas that we use as diversity measures.

#### Richness metrics

The simplest measure of diversity is species richness, that is, the number of species observed per sampling unit. However, this metric can only be used safely when the applied counting approach ensures high species detectability.

In case of phytoplankton, species detectability depends strongly on counting effort, therefore, measures that are standardised by the number of individuals observed, e.g. Margalef and Mehinick indices (Clifford & Stephenson, 1975) safeguard against biased interpretations. Ideally, standardization should take place in the process of identification. Pomati et al. (2015) gave an example how a general detection limits could be applied in retrospect to data stemming from variable counting efforts.

Species richness can also be given using richness estimators. These can be parametric curve-fitting approaches, non-parametric estimators, and extrapolation techniques using species accumulation or species-area curves (Gotelli & Colwell, 2011; Magurran, 2004). These approaches have been increasingly applied in phytoplankton ecology (Naselli-Flores et al., 2016; Görgényi et al., 2019).

#### Abundance-based metrics

Classical diversity metrics such as Shannon and Simpson indices combine richness and evenness into univariate vectors. Though used commonly in the literature, they are prone to misinform about the actual changes in a community, as they may reflect changes in evenness and/or richness to an unknown extent (a change in Shannon H' 1948) may solely be driven by a change in evenness or richness). Dominance metrics emphasise the role of the most important species (McNaughton, 1967). Rarity metrics, in contrast, focus on the rare elements of the assemblages (Gotelli & Colwell, 2011).

Species abundance distributions (SAD) and rank abundance distributions (RAD: ranking the species’ abundances from the most abundant to the least abundant) provide an alternative to diversity indices (Fisher et al., 1943; Magurran & Henderson, 2003). These parametric approaches give accurate information on community structure and are especially useful when site level data are compared. Most RADs follow lognormal distributions and allow to estimate species richness in samples (Ulrich & Ollik, 2005).

### Mechanisms affecting diversity

#### Community assembly

Understanding the processes that shape the community structure of phytoplankton requires some knowledge on the general rules of community assembly. Models and mechanisms, which have been proposed to explain the compositional patterns of biotic communities, can be linked together under one conceptual framework developed by Vellend (2010, 2016). Vellend proposed four distinct processes that determine species composition and diversity: speciation (creation of new species, or within-species genetic modifications), selection (environmental filtering, and biotic interactions), drift (demographic stochasticity) and dispersal (movement of individuals). The four processes interact to determine community dynamics across spatial scales from global, through regional to local. The importance of the processes strongly depends on the type of community, and the studied spatial and temporal scales (Reynolds, 1993).

Importance of evolutionary processes in the community assembly have been demonstrated by several phylogenetic ecological studies (Cavender-Bares et al., 2009) and also indicated by the emergence of a new field of science called ecophylogenetics (Mouquet et al., 2012). As far as the phytoplankton is concerned, the role of speciation can be important when the composition and diversity of algal assemblages are studied at large (global) spatial scales. However, we may note that although microscopic analyses cannot grasp it, short-term evolutionary processes do occur locally in planktic assemblages (Balzano et al., 2011; Padfield et al., 2016; Bach et al., 2018).

Demographic stochasticity influences growth and extinction risk of small populations largely (Parvinen et al., 2003; Méndez et al., 2019). Similarly, it might also act on large lake phytoplankton since population size in previous years affects the success of species in the subsequent year. Small changes in initial abundances may have strong effects on seasonal development. Demographic stochasticity, however, is crucial in small isolated waters (especially in newly created ones) where the sequence of new arrivals and small differences in initial abundances likely have a strong effect on the outcome of community assembly.

**Fig. 2:**
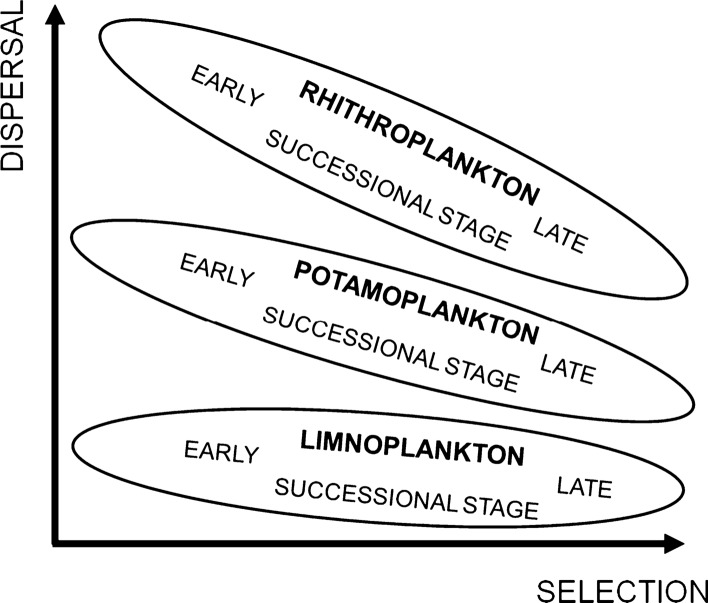
Positions of the relevant types of pelagial aquatic habitats in the selection/dispersal plane

Theoretical models, laboratory experiments and field studies demonstrated that the other two processes, selection and dispersal, have a pivotal role in shaping community assembly and diversity. Although this statement corresponds well with the Baas-Becking (1934) hypothesis (everything can be everywhere but environment selects), importance of selection and dispersal depends on the characteristics of the aquatic systems. Selection and dispersal can be considered as filters (Knopf, 1986, Pearson et al., 2018), and using them as gradients, a two-dimensional plane can be constructed, where the positions of the relevant types of pelagic aquatic habitats can be displayed (Fig. 2). At high dispersal rate, the mass effect (or so-called source-sink dynamics) is the most decisive process affecting community assembly (Leibold & Chase, 2017). Phytoplankton of rhithral rivers is a typical example of the sink populations because its composition and diversity are strongly affected by the propagule pressure coming partly from the source populations of the benthic zone and from the limnetic habitats of the watershed (Bolgovics et al., 2017). The relative importance of the mass effect decreases with time and with the increasing size of the river, while the role of selection (species sorting) increases. Due to their larger size, the impact of the source-sink dynamics in potamal rivers must be smaller, and selection becomes more important in shaping community assembly. Although the role of spatial processes in lake phytoplankton assembly cannot be ignored, their importance is considerably less than that of the locally acting selection. Relevance of the spatial processes have been demonstrated for river floodplain complexes (Vanormelingen et al., 2008; Devercelli et al., 2016; Bortolini et al., 2017), or for the lakes of Fennoscandia (Ptacnik et al., 2010a, b), where the large lake density facilitates the manifestation of spatially acting processes. High selection and low dispersal represent the position of phytoplankton inhabiting isolated lakes. Reviewing the literature of algal dispersal Reynolds concluded (2006) that cosmopolitan and pandemic distribution of algae is due to the fact that most of the planktic species effectively exploit the dispersal channels. However, he also noted that several species are not good dispersers, therefore, endemism might occur among algae.

Composition and diversity of these assemblages are controlled by the locally acting environmental filtering and by biotic interactions, frequently, by competition. The environmental filtering metaphor appears in Reynolds’ habitat template approach (Reynolds, 1998), where the template is scaled against quantified gradients of energy and resource availability. The template represents the filter, while the habitats mean the porosity (Reynolds, 2003). Species that manage to pass the filter are the candidate components of the assemblages. Finally, low-level biotic interactions (Vellend, 2016) determine the composition and diversity of the communities.

The four mechanisms, proposed by Vellend, act differently on the various metric values of diversity. Using the special cases of Rényi’s entropy (α: → 0, 1, 2, ∞) (ESM Box 1) we can show how mechanisms influence species richness and species inequalities, and how they act on the metrics between these extremes (ESM Table 1). Drivers of functional diversity are identical with that of species diversity, but their impacts are attenuated by the functional redundancy of the assemblages.

#### The role of competition in the maintenance of diversity

The concept of competition and coexistence has been first proved experimentally both for artificial two-species systems (Tilman & Kilham, 1976; Tilman, 1977) and for natural phytoplankton assemblages (Sommer, 1983). However, limitations by different nutrients are responsible only for a small portion of diversity, even if the micronutrients are also included. Therefore, it was an important step when Sommer (1984) applying a pulsed input of one key nutrient in a flow-through culture managed to maintain the coexistence of several species; although they were competing for the same resource. Several competition experiments have been carried out in recent years demonstrating the role of inter- (Ji et al., 2017) and intra-specific competition (Sildever et al., 2016) in the coexistence of planktic algae.

The fact that one single resource added in pulses can maintain the coexistence of multiple species has been also proved by mathematical modelling (Ebenhöh, 1988). Using deterministic models, Huisman & Weissing (1999) showed that competition for three or more resources result in sustained species oscillations or chaotic dynamics even under constant resource supply. These oscillations in species abundance make possible the coexistence of several species on a few limiting resources (Wang et al., 2019).

#### The non-equilibrium nature of phytoplankton and the role of disturbances

One of the underlying assumptions of the classical competition theories is that species coexistence requires a stable equilibrium point (Chesson & Case, 1986). However, the stable equilibrium state is not a fundamental property of ecosystems (DeAngelis & Waterhouse, 1987; Hastings et al., 2018). Hutchinson put forward the idea that phytoplankton diversity could be explained by “permanent failure to achieve equilibrium” (Hutchinson, 1941). On a sufficiently large timescale, ecosystems seem to show transient dynamics, and do not necessarily converge to an equilibrium state (Hastings et al., 2018). However, the virtually static equilibrium-centred view of ecological processes cannot explain the transient behaviour of ecosystems (Holling, 1973; Morozov et al., 2019). Today, there is a broad consensus in phytoplankton ecology that composition and diversity of phytoplankton can be best explicable by non-equilibrium approaches (Naselli-Flores et al., 2003). The non-equilibrium theories do not reject the role of competition in community assembly but place a larger emphasis on historical effects, chance factors, spatial inequalities, environmental perturbations (Chesson & Case, 1986), and transient dynamics of the ecosystems (Hastings, 2004). The interactions among the internally driven processes and the externally imposed stochasticity of environmental variability as an explanation of community assembly have been conceptualized in the Intermediate Disturbance Hypothesis (IDH) (Connell, 1978). This hypothesis predicts a unimodal relationship between the intensities and frequencies of disturbances and species richness. Although this hypothesis has been developed for macroscopic sessile communities, it has become widely accepted in phytoplankton ecology (Sommer, 1999). It has been proposed that the frequency of disturbances has to be measured on the scale of generation times of organisms (Reynolds, 1993; Padisák, 1994). Field observation suggested that diversity peaked at disturbance frequency of 3–5 generation times (Padisák et al., 1988), which was also corroborated by laboratory experiments (Gaedeke & Sommer, 1986; Flöder & Sommer, 1999). The IDH, however, is not without weaknesses (Fox, 2013). Recognition and measurement of disturbance are among the main concerns (Sommer et al., 1993). Diversity changes are measured purely as responses to unmeasured events (disturbances) (Juhasz-Nagy, 1993), which readily leads to circular reasoning. Repeated disturbances might change the resilience of the system, which modifies the response of communities and makes the impact of disturbances on diversity unpredictable (Hughes, 2012).

#### Amalgamation of the equilibrium and non-equilibrium concepts

The existence of the equilibrium and non-equilibrium explanations of species coexistence represents a real dilemma in ecology. Being sufficiently different, and thus avoid strong competition, or sufficiently similar with ecologically irrelevant exclusion rates (as it is suggested by Hubbell’s neutral theory (2006)) are both feasible strategies for species (Scheffer & van Nes, 2006). Coexistence of species with these different strategies is also feasible if the many sufficiently similar species create clusters along the niche axes (in accordance with Hubbel’s (2006) neutral theory), and the competitive abilities within the clusters are sufficiently large. It has been demonstrated that the so-called “lumpy coexistence” is characteristic for phytoplankton assemblages (Graco-Roza et al., 2019). Lumpy coexistence arises in fluctuating resource environments (Sakavara et al., 2018; Roelke et al., 2019), and show higher resilience to species invasions (Roelke & Eldridge, 2008) and higher resistance to allelopathy (Muhl et al., 2018).

The model of lumpy coexistence has its roots in mechanistic modelling of species coexistence (Scheffer & van Nes, 2006). Analysing lake phytoplankton data Reynolds (1980, 1984, 1988) demonstrated that species with similar preferences and tolerances to environmental constraints like nutrients or changes in water column stratification frequently coexist. These empirical observations were formalised later in the functional group (FG) concept (Reynolds et al., 2002). Despite their different theoretical backgrounds, the two approaches came to identical conclusions: species having similar positions on the niche axes form species clusters (or FGs), and in natural assemblages clusters or FGs coexist. Thus, the concept of lumpy coexistence can also be considered as a mechanistic explanation of the Reynolds’s FG concept.

The mechanisms and forces detailed above can explain how diversity is maintained at the local scale. Recent metacommunity studies, however, indicate that spatial processes have a crucial role in shaping phytoplankton diversity (Devercelli et al., 2016; Bortolini et al., 2017; Guelzow et al., 2017; Benito et al., 2018). Despite the increasing research activity in this field, spatial processes are far less studied than local ones. More in-depth knowledge on the role of connectivity of aquatic habitats and dispersal mechanisms of the phytoplankters will contribute to better understand phytoplankton diversity at regional or global scales.

### Changes of diversity along environmental scales

#### Species–area relationships across systems

The area dependence of species richness deserved special attention in ecology both from theoretical and practical points of view. The increase of species number with the area sampled is an empirical fact (Brown & Lomolino, 1998). The first model that described the so-called species–area relationship (SAR) appeared first by Arrhenius (1921) who proposed to apply power law for predicting species richness from the surveyed area. Because of the differences in the studied size scale and the studied organism groups, several other models have also been proposed such as the exponential (Gleason, 1922), the logistic (Archibald, 1949) and the linear (Connor & McCoy, 1979) models. However, the power-law (*S* = *c* × *A*^*z*^, where *S*: number of species; *A*: area sampled; *c*: the intercept, *z*: the exponent) is still the most widely used formula in SAR studies. The rate of change of the slope with an increasing area (*z* value) depends on the studied organisms, and also on the localities. High values (*z*: 0.1–0.5) were reported for macroscopic organisms (Durrett & Levin, 1996), while low *z* values characterised (*z*: 0.02–0.08) the microbial systems (Azovsky, 2002; Green et al. 2004; Horner-Devine et al. 2004).

The phytoplankton SAR appeared first in Hutchinson’s (1961) paper, where he analysed Ruttner’s dataset on Indonesian (Ruttner, 1952), and Järnefelt’s (1956) data on Finnish lakes. He concluded that there was no significant relationship between the area and species richness. Hutchinson reckoned that contribution of the littoral algae to the phytoplankton might be relevant, and because the littoral/pelagic ratio decreases with lake size, this contribution also decreases. Therefore, species richness cannot increase with lake area. In a laboratory experiment, Dickerson & Robinson (1985) found that large microcosms had significantly smaller species richness values than small ones. Based on laboratory studies, published species counts from ponds lakes and oceans, Smith et al. (2005) studied phytoplankton SAR in the possible largest size scale (10^−9^ to 10^7^ km^2^). They demonstrated a significant positive relationship between area and species richness. The calculated *z* value (*z* = 0.134) was higher than those reported in other microbial SAR studies. However, we note that this study suffers from a methodological shortcoming, because of differences in compilation of species inventories. Therefore, the results are only suggestive of possible trends that should be investigated more thoroughly.

Analysing phytoplankton monitoring data of 540 lakes in the USA Stomp et al. (2011) found only a slight increase in richness values with a considerable amount of scatter in the data. The covered size range was small in this study, and the applied counting techniques could lead to bias in richness estimation. Phytoplankton species richness showed a similar weak relationship with lake size for Scandinavian lakes (Ptacnik et al., 2010a, b), although the counting effort was much better standardised. All the above studies suggested that species richness was not independent of water body size. However, because of the methodological differences, and differences in the covered water body size, in richness estimation or the type of the water bodies, any conclusions based on these results should be handled with caution.

Nutrients, latitudinal and altitudinal differences (Stomp et al., 2011) or the size of the regional species pool (Fox et al., 2000, Ptacnik et al., 2010a, b) also influence phytoplankton diversity. To reduce the impact of these factors, Várbíró et al. (2017) investigated phytoplankton SAR in a series of standing waters within the same ecoregion and with similar nutrient status. The water bodies covered ten orders of magnitude size range (10^−2^ to 10^8^ m^2^). In this study, both the sampling effort and the sample preparation was standardised. The authors demonstrated that species richness did not scale monotonously with water body size. They managed to show the presence of the so-called Small Island Effect (SIE, Lomolino & Weiser, 2001), the phenomenon, when below a certain threshold area (here 10^−2^ to 10^2^ m^2^ size range) species richness varies independently of island size. A right-skewed hump-shaped relationship was found between the area and phytoplankton species richness with a peak at 10^5^–10^6^ m^2^ area. This phenomenon has been called as Large Lake Effect (LLE) by the authors, and they explained it by the strong wind-induced mixing, which acts against habitat diversity in the pelagic zones of large lakes. The significance of this study is that its results help explain the controversial results of earlier phytoplankton SAR studies. The LLE explains why the species richness had not grown in the case of the Ruttner’s and Jarnefelt’s dataset. The SIE, however, explains why Dickerson & Robinson (1985) found opposite tendencies to SAR in microcosm experiments. Detailed analysis of the phytoplankton in those water bodies that produced the peak in the SAR curve in the study of Várbíró et al. (2017) demonstrated that high diversity has been caused by the intrusion of metaphytic elements to the pelagic zone (Görgényi et al., 2019), which can be considered as a within-lake metacommunity process.

#### Productivity–diversity relationships

Despite the more than half a century-long history of investigations on the productivity/diversity relationship (PDR), the shape of the relationship and the underlying mechanisms still remain a subject of debate. The models describing the PDR vary from the monotonic increasing, through the hump shaped and u-shaped to the monotonic decreasing types in the literature (Waide et al., 1999). In the PDR studies, there are great differences in the applied scale (local/regional/global), in the metric used to define productivity (e.g., nutrients, biomass, production rate, precipitation, evaporation), in the used diversity metrics, and also in the studied group of organisms (special phylogenetic groups, functional assemblages) (Mittelbach et al., 2001). PDR studies also have other methodological and statistical problems (Mittelbach et al., 2001). These differences in approaches may generate different patterns, which lead to confusion and inconclusive results (Whittaker & Heegaard, 2003; Hillebrand & Cardinale, 2010). Despite these uncertainties, the most general PDR patterns are the hump-shaped and positive linear relationships; the first has been observed mostly in the case of local, while the second in the case of regional scale studies (Chase & Leibold, 2002; Ptacnik et al., 2010a). These patterns are so robust that they have been shown for various organisms independently from the highly different proxies applied to substitute the real productivity.

The number of studies that explicitly focus on phytoplankton PDR is few. The view that phytoplankton diversity peaks at intermediate productivity level has been demonstrated by several authors (Ogawa & Ichimura, 1984; Agustí et al., 1991; Leibold, 1999). This is greatly due to the fact that phytoplankton studies fortunately do not suffer from scaling problem: most studies use sample-based local *α*–*s* as diversity metrics and nutrients or biomass (Chl-a) as a surrogate measure of productivity. Unimodal relationships were found for Czech (Skácelová & Lepš, 2014) and Hungarian water bodies (Borics et al., 2014). Diversity peaked in both cases at the 10^1^–10^2^ mg L^−1^ biovolume range, characteristic for eutrophic lakes.

It has also been demonstrated that the unimodal relationship was also true for the functional richness/productivity relationship (Borics et al., 2014; Török et al., 2016). Differences were also found between the species richness and functional richness peaks; the latter peaked at smaller biovolume range (Török et al., 2016). We note here that all three studies were based on monitoring data, and because of the applied sample processing, species richness values might be slightly underestimated.

Several theories have been proposed to explain this unimodal pattern. Moss (1973) reckoned that the relationship could be accounted for by that the populations of oligotrophic and eutrophic lakes overlapping at the intermediate productivity range. Rosenzweig’s (1971) paradox of enrichment hypothesis explained the unimodal relationship by the destabilized predator–prey relationship at enhanced productivity level. Tilman’s resource heterogeneity model (1985) predicts that the coexistence of competing species is enhanced when the supply of alternative resources is heterogeneous both spatially and temporally. This heterogeneity increases with resource supply together with species richness up to the point when richness declines because the correlation between spatiotemporal heterogeneity and resource supply disappeares. The resource-ratio hypothesis can also provide an explanation of the hump shaped PDR (Tilman & Pacala, 1993; Leibold, 1997). This theory suggests that relative supply of resources generates variations in species composition. Identity of the most strongly limiting resource changes, and at very high resource supply (on the descending end of the curve) only a few K-strategist specialists will prevail. The species pools overlap at intermediate productivity level, resulting in high species richness. This explanation seems to be reasonable for phytoplankton PDR studies.

Investigating the PDR in fishless ponds, Leibold (1999) found that his results could be best explained by the keystone predation hypothesis (Paine, 1966). This theory asserts that at low productivity exploitative competition is the main assembly rule, while with increasing productivity range the role of predator avoidance becomes more important.

The number of various explanations illustrates the complexity of processes affecting the shape of the PDR. The shifting effects of bottom-up *vs.* top-down control on the trophic gradient, the size of the regional species pool, that is, the number of potential colonizers, or the history of the studied water bodies (naturally eutrophic lakes are studied, or eutrophicated formerly oligotrophic ones) can considerably modify the properties of the PDRs.

With a few exceptions (Irigoien et al., 2004), phytoplankton PDRs have been studied almost exclusively in standing waters.

Investigating the phytoplankton PDRs in rivers Borics et al. (2014) found monotonic increasing pattern in rhithral and monotonic decreasing PDR in potamal rivers. They explained the positive linear PDR with the newly arriving species from the various adjacent habitats of the watershed, which resulted in high phytoplankton diversity even at highly eutrophic conditions. This phytoplankton is a mixture of those elements that enter the river from the connected water bodies of various types. In contrast, potamal rivers are highly selective environments in which the phytoplankton succession frequently terminates in low diversity plankton dominated by K strategist centric diatoms (*Cyclotella* and S*tephanodiscus* spp.).

We note here that study of the regional phytoplankton PDR should be an important and challenging area of future work, which is presently hindered by the disconnected databases and by difficulties in measuring regional productivity.

#### Linkage between diversity and the metabolic theory of ecology

Metabolism controls patterns, processes and dynamics at each level of biological organisation from single cells to ecosystems, summarised as the metabolic theory of ecology (Brown et al., 2004). Metabolic theory (MTE) provides alternative explanations for observations on various fields of ecology such as in individual performance, life history, population and community dynamics, as well as in ecosystem processes. According to MTE, dynamics of metabolic processes have implications for species diversity. Metabolic processes influence population growth and interspecific competition, might accelerate evolutionary dynamics and the rate of speciation (Brown et al., 2004). The direct linkage between temperature and metabolic rate raises the possibility of new explanations of the well-known latitudinal dependence of species richness. Allen et al. (2002) found that for both terrestrial and aquatic environments natural logarithm of species richness should be a linear function of the mean temperature of the environment. This model has been tested both for lake and oceanic phytoplankton. Investigating more than 600 European, North and South American lakes Segura et al. (2015) found a pronounced effect of temperature on species diversity between 11 and 17 °C. Righetti et al. (2019) analysed the results of more than 500,000 phytoplankton observations from the global ocean, and also showed the relationship between temperature and species richness, but similarly to freshwater lakes the relationship was not monotonic for the whole temperature gradient. These results suggest that the MTE can be a possible explanation for the temperature dependence of diversity. However, we note that other theories emphasising longer “effective” evolutionary time (Rohde, 1992) or higher resource availability (Brown & Lomolino, 1998) can also explain this general pattern.

### The functional diversity–ecosystem functioning relationship in phytoplankton

More diverse communities perform better in terms of resource use and ecosystem stability (Naeem & Li, 1997); known as the biodiversity-ecosystem functioning relationship (BEF). Similar to BEF relationships shown in terrestrial plant communities (Tilman et al., 1996, 1997), positive BEF relationships have also been evidenced in both natural and synthetic phytoplankton communities (Ptacnik et al., 2008; Striebel et al., 2009; Stockenreiter et al., 2013). The BEF relationship itself, however, does not explain the mechanisms underlying the relationship. The most often recognised mechanisms are complementarity (Loreau & Hector, 2001) and sampling effect (Fridley, 2001). Complementarity means that more diverse communities complement each other in resource use in a more efficient way. Sampling effect, on the other hand, means that the chance increases for the presence of species with effective functional attributes in more diverse communities (Naeem & Wright, 2003).

In an attempt to get mechanistic understanding of diversity-functioning relationships, there is a growing interest in quantifying functional diversity of ecological communities (Hillebrand & Matthiessen, 2009). Functional diversity summarizes the values and ranges of traits that influence ecosystem functioning (Petchey & Gaston, 2006). By translating taxonomic into functional diversity, we may eventually also distinguish complementarity from sampling effect.

In phytoplankton ecology, two functional perspectives have been developing. First, the identification of morphological, physiological and behavioural traits (Weithoff, 2003; Litchman & Klausmeier, 2008) that affect fitness (Violle et al., 2007) and are, therefore, functional traits. Traits have been used in phytoplankton ecology at least since Margalef’s ‘life forms’ concept (Margalef, 1968; 1978), even if they were not referred to ‘traits’ explicitely (Weithoff & Beisner, 2019). Second, the recognition of characteristic functional units within phytoplankton assemblages led to the development of functional group (ecological groups) concepts (see Salmaso et al., 2015). These are the phytoplankton functional group concept sensu Reynolds (FG, Reynolds et al., 2002), the morpho-functional group concept (MFG, Salmaso & Padisák, 2007), and the morphological group concept (MBFG, Kruk et al., 2011).

The functional trait concept has been advocated in trait-based models (Litchman et al., 2007) and aimed at translating biotic into functional diversity, which eventually would allow quantify functional diversity at the community level. The functional trait concept has recently been reviewed in context of measures and approaches in marine and freshwater phytoplankton (Weithoff & Beisner, 2019). On the other hand, the ‘functional group’ concepts have rather been developed in the context of describing characteristic functional community compositions in specific set of environment conditions (that is, the functional community–environment relationship).

The simplest functional diversity measure of phytoplankton is the number of ‘functional units’ in assemblages. That is, either the number of unique combinations of functional traits or the number of ecological groups indentified. One way to use functional units is to convert them into univariate measures corresponding to those calculated from taxonomic information (e.g., richness, evenness). Or, trait data also allow the calculation of community-level means of trait values (CWM) as an index of functional community composition (Lavorel et al., 2008). Second, one may consider calculate the components of functional diversity (FD) such as functional richness, functional evenness, and functional divergence (Mason et al., 2005); all representing independent factes of functional community compositions. The same FD concept has been developed further accounting also for the abundance of taxa within a multidimensional trait space based on functional evenness, functional divergence and functional dispersion (Laliberté & Legendre, 2010). The recently developed ‘FD’ R package enables one to calculated easily all the aforementioned FD measures (Laliberté & Legendre, 2010; Laliberté et al., 2014). The use of FD components in the context of BEF in phytoplankton has only started very recently (Abonyi et al., 2018a, b; Ye et al., 2019). Trait-based functional diversity measures in BEF have recently been reviewed by Venail (2017).

#### The functional community composition–environment relationship

Functional traits can be classified as those affecting fitness via growth and reproduction (i.e., functional effect traits) and those responding to alterations in the environment (i.e., functional response traits) (Hooper et al., 2002, 2005; Violle et al., 2007). Since many ecophysiological traits, such as nutrient and light utilization and grazer resistance, correlate with phytoplankton cell size (Litchman & Klausmeier, 2008), size has been recognized as a master trait. Phytoplankton cell size responds to alterations in environmental conditions, like change in water temperature (Zohary et al., 2020), and also affects ecosystem functioning (Abonyi et al., 2020). The response of freshwater phytoplankton size to water temperature changes seems to be consequent based on both the cell and colony (filament) size (Zohary et al., 2020). However, one may consider that cell and colony (filament) sizes are affected by multiple underlying mechanisms, and the choose of cell or colony size as functional trait might be question specific.

The functional group (ecological group) composition of phytoplankton can be predicted well by the local environment (Salmaso et al., 2015). However, the different functional approaches have rarely been compared in terms of how they affect the community composition–environment relationship. Kruk et al. (2011) showed that the morphological group (MBFG) composition of phytoplankton could be predicted from the local environment in a more reliable way than Reynolds’s functional groups (FG), or taxonomic composition. In a broad-scale phytoplankton dataset from Fennoscandia, Abonyi et al. (2018a, b) showed that phytoplankton functional trait categories, as a community matrix, corresponded with the local environment better than Reynolds’s functional groups or the taxonomic matrix. Along the entire length of the Atlantic River Loire, Abonyi et al. (2014) showed that phytoplankton composition based on Reynolds’s FG classification provided more detailed correspondence to natural- and human-induced changes in environmental conditions than based on the morpho-functional (MFG) and morphological (MBFG) systems.

The aggregation of taxonomic information into functional units reduces data complexity that could come along with reduced ecological information (Abonyi et al., 2018a, b). Reduced data complexity can be useful as long as it does not imply serious loss of ecological information. Information lost can happen when functional traits are not quantified adequately, cannot be identified, or when ecologically diverse taxa, such as benthic diatoms are considered similar functionally (Wang et al., 2018). Otherwise, the aggregation of taxonomic to functional data highlights ecological similarities among taxa (Schippers et al., 2001) and should lead to better correspondence between community composition and the environment (Abonyi et al., 2018a, b).

#### The functional diversity–ecosystem functioning relationship

Based on taxonomic data, recent studies support a positive biodiversity–ecosystem functioning relationship in phytoplankton clearly (Naeem & Li, 1997; Ptacnik et al., 2008; Striebel et al., 2009). The well-known paradox of Hutchinson asking how so many species may coexist in phytoplankton (Hutchinson, 1961) has been reversed to how many species ensure ecosystem functioning (Ptacnik et al., 2010b). Based on functional traits, however, almost half of the studies reported null or negative relationship between functional diversity and ecosystem functioning (Venail, 2017). Recently, Abonyi et al. (2018a, b) argued that functional diversity based on trait categories (i.e., functional trait richness—FTR) and Reynolds’ ecological groups (i.e., functional group richness—FGR) represented different aspects of community organisation in phytoplankton. While both functional measures scaled with taxonomic richness largely, FTR suggested random or uniform occupation of niche space (Díaz & Cabido, 2001), while FGR more frequent niche overlaps (Ehrlich & Ehrlich, 1981), and therefore, enhanced functional redundancy (Díaz & Cabido, 2001). A key future direction will be to understand mechanisms responsible for the co-occurrence of functional units (‘functional groups’) within phytoplankton assemblages, and detail phytoplankton taxa within and among the ecological groups in a trait-based approach. This will enhance our ability to disentangle the ecological role of functional redundancy (within groups) and complementarity (among groups) in affecting ecosystem functioning in the future.

### Phytoplankton diversity using molecular tools

The assessment of phytoplankton diversity in waterbodies is strongly dependent from the methods used in the taxonomic identification of species and the quantitative estimation of abundances. The adoption of different methods can strongly influence the number of taxa identified and the level of detail in the taxonomic classifications.

#### Premise: advantages and weaknesses of light microscopy

Traditionally, phytoplankton microorganisms have been identified using light microscopy (LM). The use of this technique was instrumental to lay the foundation of phytoplankton taxonomy. Many of the most important and well-known species of nano- (2–20 μm), micro- (20–200 μm) and macrophytoplankton (> 200 μm) have been identified by several influential papers and manuals published between the first half of the 1800 s and first half of 1900 s (e.g. (Ehrenberg, 1830; de Toni, 1907; Geitler & Pascher, 1925; Guiry & Guiry, 2019). LM is an inexpensive method providing plenty of information on the morphology and size of phytoplankton morphotypes, allowing also obtaining, if evaluated, data on abundances and community structure. Conversely, in addition to being time-consuming, the correct identification of specimens by LM requires a deep knowledge of algal taxonomy. Further, many taxa have overlapping morphological features so that the number of diacritical elements often is not enough to discriminate with certainty different species (Krienitz & Bock, 2012; Whitton & Potts, 2012; Wilmotte et al., 2017). The identification can be further complicated by the plasticity that characterise a number of phenotypic characteristics and their dependence from environmental conditions (Komárek & Komárková, 2003; Morabito et al., 2007; Hodoki et al., 2013; Soares et al., 2013). The adoption of electron microscopy for the study of ultra-structural details has represented an important step in the characterization of critical species (e.g. Komárek & Albertano, 1994) and phyla. For example, in the case of diatoms, scanning electron microscopy had a huge impact on diatom taxonomy, making traditional LM insufficient for the recognition of newly created taxa (Morales et al., 2001). Since aquatic samples usually contain many small, rare and cryptic species, a precise assessment of the current biodiversity is unbearable with the only use of classic LM (Lee et al., 2014) and electron microscopy. Nonetheless, despite its limitations, the analysis of phytoplankton by LM still continues to be the principal approach used in the monitoring of the ecological quality of waters (Hötzel & Croome, 1999; Lyche Solheim et al., 2014).

#### Culture-dependent approaches—classical genetic characterization of strains

Owing to the above limitations, the identification of phytoplankton species by LM has been complemented by the adoption of genetic methods. These methods are based on the isolation of single strains, their cultivation under controlled conditions, and their characterization by polymerase chain reaction (PCR) and sequencing of specific DNA markers able to discriminate among genera and species, and sometimes also between different genotypes of a same species (Wilson et al., 2000; D’Alelio et al., 2013; Capelli et al., 2017). After sequencing, the DNA amplicons obtained by PCR can be compared with the sequences deposited in molecular databases, e.g. those included in the International Nucleotide Sequence Database Collaboration (INSDC: DDBJ, ENA, GenBank) using dedicated tools, such as BLAST queries (Johnson et al., 2008). Further, the new sequences can be analysed, together with different homologous sequences, to better characterize the phylogenetic position and taxonomy of the analysed taxa in specific clades (Rajaniemi et al., 2005; Krienitz & Bock, 2012; Komárek et al., 2014). The phylogenetic analyses provide essential information also for evaluating the geographical distribution of species (Dyble et al., 2002; Capelli et al., 2017) and their colonization patterns (Gugger et al., 2005), to infer physiological traits (Bruggeman, 2011), and to evaluate relationships between phylogeny and sensitivity to anthropogenic stressors in freshwater phytoplankton (Larras et al., 2014). The selection of primers and markers, and their specificity to target precise algal groups is an essential step, which strictly depends on the objectives of investigations and availability of designated databases. For example, though 16S and 18S rRNA genes are the most represented in the INSDC databases, dedicated archives have been curated for the blast and/or phylogenetic analyses of cyanobacteria (e.g. Ribosomal Database Project; Quast et al., 2013; Cole et al., 2014) and eukaryotes (e.g. Quast et al., 2013; Rimet et al., 2019). Further, an increase in the sensitivity of the taxonomic identification based on DNA markers can be obtained through the concurrent analysis of multiple genes using Multilocus Sequence Typing (MLST) and Multilocus Sequence Analysis (MLSA) (see Wilmotte et al., 2017, for details).

A potential issue with the single use of only microscopy or genetic methods is due to the existence of genetically almost identical different morphotypes and to the development of uncommon morphological characteristics in strains cultivated and maintained in controlled culture conditions. To solve these problems, a polyphasic approach has been proposed, which makes use of a set of complementary methods, based besides genetics, on the analysis of phenotypic traits, physiology, ecology, metabolomics and other characters relevant for the identification of species of different phyla (Vandamme et al., 1996; Komárek, 2016; Salmaso et al., 2017; Wilmotte et al., 2017).

Considering the existence of different genotypes within a single species (D’Alelio et al., 2011; Yarza et al., 2014), the genetic characterizations of phytoplankters have to be performed at the level of single strain. Excluding single cell sequencing analyses (see below), the methods have to be therefore applied to isolated and cultivated strains. This represents a huge limitation for the assessment of biodiversity, because the analyses are necessarily circumscribed only to the cultivable organisms. The rarest and the smaller ones are equally lost. Further, the genetic and/or the polyphasic approaches are time-consuming, allowing to process only one species at a time. To solve this limitation, a set of culture-independent approaches to assess biodiversity in environmental samples have been developed since the 1980s.

#### Culture independent approaches—traditional methods

A consistent number of molecular typing methods based on gel electrophoresis and a variety of other approaches (e.g. quantitative PCR-qPCR) have been applied since the 1980 s and 1990 s in the analysis of microbial DNA, including “phytoplankton” (for a review, see Wilmotte et al., 2017). These approaches are tuned to target common regions of the whole genomic DNA extracted from water samples or other substrata, providing information on the existence of specific taxonomic and toxins encoding genes (Campo et al., 2013; Capelli et al., 2018), and the taxonomic composition of the algal community without the need to isolate and cultivate individual strains. In this latter group of methods, probably one of the most used in phytoplankton ecology is the denaturing gradient gel electrophoresis (DGGE; (Strathdee & Free, 2013). Taking advantage of the differences in melting behaviours of double-stranded DNA in a polyacrylamide gel with a linear gradient of denaturants, DGGE allows the differential separation of DNA fragments of the same length and different nucleotide sequences (Jasser et al., 2017). This technique is able to discriminate differences in single-nucleotide polymorphisms without the need for DNA sequencing, providing information at level of species and genotypes. For example, analysing samples from eight lakes of different trophic status, Li et al. (2009) identified complex community fingerprints in both planktic eukaryotes (up to 52 18S rDNA bands) and prokaryotes (up to 59 16S rDNA bands). If coupled with the analyses of excised DNA bands (Callieri et al., 2007), or with markers composed of cyanobacterial clone libraries (Tijdens et al., 2008), DGGE can provide powerful indications on the diversity and taxonomic composition of phytoplankton. More recent examples of the application of this technique to phytoplankton and eukaryotic plankton are given in Dong et al. (2016), Batista & Giani (2019). A recent comparison of DGGE with other fingerprint methods (Terminal restriction fragment length polymorphism, TRFLP) was contributed by Zhang et al. (2018).

A second method that has been used in the characterization of phytoplankton from microbial DNA is fluorescence in situ hybridization (FISH), and catalysed reporter deposition (CARD)-FISH (Kubota, 2013). In freshwater investigations, this technique has been used especially in the evaluation of prokaryotic communities (Ramm et al., 2012). A third method deserving mention is cloning and sequencing (Kong et al., 2017).

In principle, compared to LM and traditional genetic methods, these techniques can provide an extended view of freshwater biodiversity. Nevertheless, they suffer from several limitations, due to the time, costs and expertise required for the analysis, and the incomplete characterization of biodiversity due to manifest restrictions in the methods (e.g. finite resolution of gel bands in DGGE and number and sensitivity of markers to be used in CARD-FISH). Part of these limits have been solved with the adoption of new generation methods based on the analysis of environmental and microbial DNA.

#### Culture independent approaches—metagenomics

The more modern methods boost the sequencing approach over the traditional constraints, allowing obtaining, without gel-based methods or cloning, hundreds of thousands of DNA sequences from environmental samples using high throughput sequencing (HTS). Under the umbrella of metagenomics, we can include a broad number of specialized techniques focused on the study of uncultured microorganisms (microbes, protists) as well as plants and animals via the tools of modern genomic analysis (Chen & Pachter, 2005; Fujii et al., 2019). The methods based on HTS analysis of microbial DNA can be classified under two broad categories, i.e. studies performing massive PCR amplification of certain genes of taxonomic or functional interest, e.g. 16S and 18S rRNA (marker gene amplification metagenomics), and the sequence-based analysis of the whole microbial genomes extracted from environmental samples (full shotgun metagenomics) (Handelsman, 2009; Xia et al., 2011). While full shotgun metagenomics techniques were used in the first global investigations of marine biodiversity (Venter et al., 2004; Rusch et al., 2007; Bork et al., 2015), the use of marker gene amplification metagenomics in the study of freshwater phytoplankton has shown an impressive increase in the last decade. The reasons are still due to the minor costs (a few tens of euros per sample) and the simpler bioinformatic tractability of sequences of specific genes compared to full shotgun metagenomics.

The large progress and knowledge obtained in the study of microbial communities (Bacteria and Archaea) based on the analysis of the 16S rDNA marker in the more disparate terrestrial, aquatic and host-organisms’ habitats (e.g. gut microbial communities) had a strong influence in directing the type of investigations undertaken in freshwater environments. At present, the majority of the investigations in freshwater habitats are focused on the identification of microbial (including cyanobacteria) communities, with a minority of studies focused on the photosynthetic and mixotrophic protists (phytoplankton) evaluated through deep sequencing of the 18S rDNA marker (e.g. (Mäki et al., 2017; Li & Morgan-Kiss, 2019; Salmaso et al., 2020).

The results obtained from the applications of HTS to freshwater samples are impressive and are unveiling a degree of diversity in biological communities previously unimaginable, including a significant presence of the new group of non-photosynthetic cyanobacteria (Shih et al., 2013, 2017; Salmaso et al., 2018; Monchamp et al., 2019; Salmaso, 2019). Nonetheless, the application of these techniques is not free from difficulties, due to (among the others) the semiquantitative nature of data, the short DNA reads obtained by the most common HTS techniques, the variability in the copy number per cell of the most common taxonomic markers used (i.e. 16S and 18S rDNA), the incompleteness of genetic databases, which are still fed by information obtained by the isolation and cultivation approaches (Gołębiewski & Tretyn, 2020; Salmaso et al., 2020). Despite these constraints, the use of HTS techniques in the study of phytoplankton, which is just at the beginning, is contributing to revolutionize the approach we are using in the assessment of aquatic biodiversity in freshwater environments, opening the way to a next generation of investigations in phytoplankton ecology and a new improved understanding of plankton ecology.

## Conclusions

In this study, we reviewed various aspects of phytoplankton diversity, including definitions and measures, mechanisms maintaining diversity, its dependence on productivity, habitat size and temperature, functional diversity in the context of ecosystem functioning, and molecular diversity.

Phytoplankton diversity cannot be explained without the understanding of mechanisms that shape assemblages. We highlighted how Vellend’s framework on community assembly (speciation, selection, drift, dispersal) could be applied to phytoplankton assemblages. Competition theories and non-equilibrium approaches fitted also well into this framework.

The available literature on phytoplankton species–area relationship contains information on isolated habitats. These studies argue that richness depends on habitat size. However, findings on eutrophic shallow water bodies suggest that habitat diversity can modify the monotonous increasing tendencies and hump-shaped relationship might occur. The literature on lake’s phytoplankton productivity–diversity relationship supports trends reported for terrestrial ecosystems, i.e. a humped shape relationship at local scale if a sufficiently large productivity range is considered. However, the shapes of the curves depend also on the types of the water bodies. In rivers, both monotonic increasing (rhithral rivers) and decreasing (potamal rivers) trends could be observed.

The aggregation of phytoplankton taxonomic data based on functional information reduces data complexity largely. The reduced biological information could come along with ecological information loss, e.g. when traits cannot be quantified adequately, or, when ecologically diverse taxa are considered similar functionally. Since pelagic phytoplankton is relatively similar functionally, the aggregation of taxonomic into functional data can highlight ecological similarities among taxa in a meaningful way. Accordingly, functional composition and diversity may help better relate phytoplankton communities to their environment and predict the effects of community changes on ecosystem functioning.

The adoption of a new generation of techniques based on the massive sequencing of selected DNA markers and planktonic genomes is beginning to change our present perception of phytoplankton diversity. Moreover, being “all-inclusive” techniques, HTS are contributing to change also the traditional concept of “phytoplankton”, providing a whole picture not only of the traditional phytoplankton groups, but of the whole microbial (including cyanobacteria) and protist (including phytoplankton) communities. The new molecular tools not only help species identification and unravel cryptic diversity, but provide information on the genetic variability of species that determine their metabolic range and unique physiological properties. These, basically influence speciation and species performances in terms of biotic interactions or colonisation success, and thus affect species assembly.

## Outlook

Overexploitation of ecosystems and habitat destructions coupled with global warming resulted in huge species loss on Earth. The rate of diversity loss is so high that scientists agree that the Earth’s biota entered the sixth mass extinction (Ceballos et al., 2015). While population shrinkage or extinction of a macroscopic animal receive large media interest (writing this sentence we have the news that the Chinese paddlefish/*Psephurus gladius/*declared extinct), extinction rate of poorly known taxa can be much higher (Régnier et al., 2015). Phytoplankton, invertebrates and microscopic organisms belongs to groups where extinctions do occur, but the rate of extinctions cannot be assessed. Worldwide, thousands of phytoplankton samples are investigated every day, mostly for water quality monitoring purposes. However, assessment methods focus on the identification of the dominant and subdominant taxa, because these determine mostly the values of quality metrics. Since species richness or abundance-based diversity metrics are not considered as good quality indicators (Carvallho et al., 2013), investigators are not forced to reveal the overall species richness of the samples. To give an accurate prediction for the species richness of a water body, an extensive sampling strategy and the use of species estimators would be required. Nevertheless, high local species richness does not necessarily mean good ecosystem health and high nature conservation value; e.g. if weak selection couples with high number of new invaders. Small water bodies with low local alpha diversity but with unique microflora can have high conservation value (Bolgovics et al., 2019). Preservation of large phytoplankton species diversity at the landscape or higher geographic level needs to maintain high beta diversity by the protection of unique habitats (Noss, 1983). Because of the multiple human impacts and global warming, small water bodies belong to the most endangered habitats whose protection is of paramount importance.

Our understanding about phytoplankton diversity has progressed in the recent decades. These were mainly motivated by elucidating mechanisms that drive diversity, and by the emergence of new approaches for analysing relationships between diversity and ecosystem functioning.

Increasing human pressure and global warming-induced latitudinal shifts in climate zones, resulting in hydrological regime shifts with serious implications for aquatic ecosystems including phytoplankton. These timely challenges will also affect near future trends in phytoplankton studies. The sound theoretical principles, together with the new molecular and statistical tools open new perspectives in diversity research, which, may let us hope that the Golden Age of studying phytoplankton diversity lies before us and not behind.

## Dedication

Each study in this special issue of Hydrobiologia is dedicated to the memory of the late Colin S. Reynolds, who made an outstanding contribution to aquatic science, and considered one of the most prominent phytoplankton ecologists of the last three decades. His encyclopedic work, *The ecology of phytoplankton* (2006) considered by many as the Bible for lake phytoplankton ecology, and serves still as a reference for many recent works. His oeuvre covers a wide range of topics within aquatic ecology, including community assembly, functional approaches, modelling of biomass production, resilience and health of aquatic ecosystems. Reynolds’s contribution to our understanding of diversity maintenance mechanisms is still relevant and served as a basis for shaping our manuscript.

## Electronic supplementary material

Below is the link to the electronic supplementary material.

Supplementary material 1 (DOCX 25 kb)
