# Supplementary material for: Freshwater phytoplankton diversity: models, drivers and implications for ecosystem properties
Source: Hydrobiologia. 2020 Jul 4;848(1):53–75. doi: 10.1007/s10750-020-04332-9 (PMC7334633; doi:10.1007/s10750-020-04332-9)
Supplement: Supplementary file 1 — Supplementary material 1 (DOCX 25 kb) [file 10750_2020_4332_MOESM1_ESM.docx]

**Electronic supplementary material**

Box 1

Rényi entropies as extensions of the Shannon’s formula

Rényi (1961) using a scale parameter (α) generalised the Shannon’s entropy (Eq. 1). This extension makes possible to display diversity relations graphically in a way that the values of Rényi’s entropy are plotted against the selected values of the scale parameter (α) (Tóthmérész, 1995). The contribution of abundant taxa to diversity increases with the increase of the value of α. When α is equal to 0, the value of the Rényi diversity is the logarithm of the number of species (Eq. 2). At α→1 Rényi diversity is identical to the Shannon index of diversity (Shannon, 1948) (Eq. 3). When the value of the scale parameter is 2, the Rényi diversity is identical to the Simpson diversity (Eq. 4). Finally, when value of α approaches to infinity (α → ∞) Rényi diversity is equivalent to Berger–Parker diversity (Berger & Parker, 1970), that is, to the log of the relative abundance of the most frequent taxon.

Eq.1 $HR_{\alpha}=\frac{1}{1-\alpha}\log\sum_{i=1}^{S} p_{i}^{\alpha}$ where α ≥0 and α ≠ 1

Eq.2 $HR_{0}=\log S$

Eq.3 $\lim_{\alpha\to1} HR_{\alpha}\equiv HR_{1}=-\sum_{i=1}^{S} p_{i}\log p_{i}$

Eq.4 $HR_{2}=-\log\sum_{i=1}^{S} \text{p}_{\text{i}}^{2}$

Eq.5 $HR_{\infty}=-\log(\max p_{i})$

Table 1. Relationships between the community assembly rules and the values of characteristic measures of Rényi diversity family (α:scale parameter of Rényi’s entropy) in the case of local assemblages. The + symbols indicate that larger intensity of the given assembly process results in an increase in the metric values. The – symbols refer to negative relationships.
